# Annotations

**Published:** 1904-06-18

**Authors:** 


### Annotations.

### Lord Balfour on Hospitals.

It is highly satisfactory to find that a statesman of such large experience and practical instincts as Lord Balfour of Burleigh is concerned with the arrangements—or, perhaps, we should say the want of them—which at present exist for the supply of medical and surgical treatment to the sick poor. In his presidential address to the International Home Relief Congress recently held in Edinburgh, Lord Balfour directed attention to this subject as one on which "a great and serious reform is needed if things are to be placed on a really sound and proper footing." This criticism, be it observed, was not restricted to Poor-law relief, but included the practice of hospitals, infirmaries, provident dispensaries, friendly society insurance, and even the work of the private practitioner amongst the humbler classes of the population. The whole of this, it seems to Lord Balfour, is in a state of hopeless disorganisation, and any scheme of reform or improvement must recognise that the work must be begun from the very foundation. There will be a general readiness to confirm these statements on the part of all those who are acquainted with the true state of matters at present existing. Crowds of out-patients may thrill the heart of the charitable public, and may delight those who have to extract from this public the means of support for our hospitals, but will anyone say that in the existing circumstances they promote either public policy or the success of scientific and practical medicine? Lord Balfour's suggestions that the hospital out-patient staff should act as consultants in cases referred to them by outside practitioners, and that deserving cases should be received into the hospital, or in the last resort passed on to the Poor-law authorities, are excellent. What is wanted is the practical man of affairs who can organise these proposals into action.

### University Degrees in Pharmacy.

The suggestion that British Universities should offer opportunities for graduation in pharmacy has been advanced on many occasions, and some two or three years ago began to take practical shape. Since that date negotiations have been in progress between the authorities of the several Scottish Universities, and it appears probable that a joint conclusion will now be reached. A draft ordinance has been framed, and has been discussed and approved generally by the Senatus of each University, but the question has yet to come before the University Courts, and behind these there yet lies a Committee of the Privy Council and the Houses of Parliament. Hence there are abundant opportunities for critics or opponents to arise and express themselves. Clearly the question is not one solely for the Universities and the pharmacists. It concerns the general public and more particularly those who hold University degrees, as the status and distinction of these are apt to be qualified by the introduction of any new order of graduates. So far as the medical profession is concerned, we believe there will be an attitude of general sympathy towards any proposal which will improve the education and scientific training of the pharmacist. In some quarters there will be a measure of anxiety lest University degrees shall become exploited for purely commercial purposes and shall be used to adorn trade advertisements of a character only too familiar to us. In so far as this fear exists pharmacists have to thank certain members of their own calling whose zeal has not always been according to knowledge; but it may be anticipated that in pharmacy, as in other instances, the discipline of a University training will check any tendency to that undue self assertion which is the note of imperfect culture and narrow education.

### An Encouraging Incident.

In several of the sermons preached on Hospital Sunday the possibility of the hospitals being thrown on the rates, was referred to and deprecated. At St. Paul's Cathedral in the afternoon the Bishop of Stepney said he had no doubt that, under public-moneyed hospitals, the doctors would show all their skill and willingness, that the nurses would show all their tenderness and patience; but the moral self respect of London was at stake, and if the hospitals were thrown on the rates, the last tie which bound the wealth of the richest of all cities to the spirit of stewardship would be snapped. A few hours earlier the Rev. Dr. Carter, vicar of St. Dionis, Fulham, told his congregation that just before morning service a letter had been sent to him from a parishioner who was not able to be present enclosing the sum of a sovereign which he wished to contribute to the Hospital Sunday Fund. The man was only in humble circumstances, a waiter in fact; but he was anxious to manifest his gratitude for the benefits which he had received from one of the great charitable institutions. This incident is a reproach to the wealthy and to the well-to-do who neglect the responsibility of their stewardship; it is a reproof to the pessimists who believe that the transfer of the hospitals to the charge of the rates is inevitable. We, of course, share to the full the opinion of the Prince of Wales, cited by the Chief Rabbi in his sermon at the Great Synagogue on Saturday, that the transfer would not only involve an immense increase in the rates, but would also shift the burden from those who are well able to bear it to those who are already overburdened. But it is inconceivable that while there is among the poor such a feeling of appreciation and such a determination to bear witness to it in a practical manner, the rich will break the tie which the Bishop of Stepney entreats them to preserve. So long as those who profit directly by the work of mercy carried on in the London hospitals make it clear that they are thankful by giving out of their hard-earned wages sums which ought to put to the blush the fashionably clothed worshipper in church or chapel who contributes a shilling to the collection, and thinks a duty has been nobly performed, we shall not relinquish the conviction that sympathy for suffering will continue to ensure the maintenance of the hospitals on the voluntary principle. If ingratitude does much to dry up the springs of compassion, gratitude exhibited in a way that cannot be mistaken tends to keep them flowing.

